# Quality of life in families under quarantine: a cross-sectional study in seven countries during the first outbreak of COVID-19

**DOI:** 10.3389/fpsyt.2023.1238569

**Published:** 2023-09-05

**Authors:** Jeel Moya-Salazar, Erika Chiu-Higa, Alexis Jaime-Quispe, Betsy Cañari, Jeel G. Moya-Espinoza, Hans Contreras-Pulache

**Affiliations:** ^1^Faculties of Health Science, Universidad Privada del Norte, Lima, Peru; ^2^Department of Medicine, Hospital Nacional Alberto Sabogal Sologuren, Lima, Peru; ^3^Qualitative Unit, Nesh Hubbs, Lima, Peru; ^4^Digital Transformation Center, Universidad Norbert Wiener, Lima, Peru

**Keywords:** COVID-19, quality of life, public health, family structure, Latin America

## Abstract

**Background:**

The COVID-19 pandemic has disrupted human well-being worldwide in unforeseen ways. In early 2020, the spread of the virus left its mark on every affected country, impacting mental health by limiting daily activities and causing fatalities amidst public health strategies to mitigate its impact. The influence of COVID-19 on the quality of life (QoL) may vary between countries, underscoring the need to examine its effects on individuals and families during the mandatory home quarantine. We aimed to assess the QoL of individuals and families during home isolation by COVID-19 lockdown.

**Methods:**

A cross-sectional study was conducted from February to May 2020. We included adult partners (≥18 years) of families from Brazil, Colombia, Spain, Japan, Peru, Russia, and Venezuela. Using the 26-item World Health Organization Quality of Life Brief (WHOQOL-BREF) questionnaire we assess the impact of COVID-19 on their partner/family member’s QoL.

**Results:**

The survey was completed by 466 participants (mean age = 38.59 ± 13.75 years; females = 298) and 76% worked mostly as health professionals from South America (69.2%), Europe (18.4%), and Asia (12.4%). The WHOQOL-BREF mean score from 38.38 ± 11.55 (range = 22.8–43.4). The average quality of life in South America (41.9 ± 1.2) was significantly higher than that of European countries (30.9 ± 11.5) (*p* = 0.002). The social relations dimension was the only one with values close to 100 (mean = 83.3) in 6/7 evaluated countries, where only Spain had a low score (41 ± 33.12). Women had a slightly lower quality of life than men, but it was not significant (40.2 ± 8.8 vs. 41.5 ± 9.9, *p* = 0.354), while we found differences in the overall QoL between young and older, and by employment type (*p* < 0.05). According to family structure, we found differences on QoL between nuclear and siblings’ families (*p* = 0.024).

**Conclusion:**

Families from seven countries of three continents reported poor QoL during the first outbreak of COVID-19. The pandemic scenario has dramatically weakened the QoL in 3/4 dimensions, where social relationships have remained high. It is important to fully address the impact of this reduced QoL on families after several waves of infection and to provide comprehensive support in the post-COVID future.

## 1. Introduction

Individual health has been dramatically affected when the COVID-19 pandemic started, due to the great extent of restrictions that each country imposed in order to reduce the spread of SARS-CoV-2. This crisis, which began in 2020, has limited human freedom due to the decrease of local and international mobility, confinement, and the prohibition of mass gatherings (1, 2). Rapid adoption of home isolation and quarantine, on the one side, has reduced infection and death increase; and, on the other hand, it has seriously affected economy worldwide (3, 4).

However, quarantines have been unequal among countries. COVID-19 has led to social isolation in short periods in several countries (5); while in others, it has extended over many months (6). Quarantines have also been subject to qualitative and quantitative factors that have impacted the comfort or discomfort during the lockdowns all around the world (7). Thus, higher-income populations with more resources and spaces have not shown significant changes in their well-being according to what some studies report (8, 9), however, a comprehensive analysis of families across different countries has not yet been conducted.

COVID-19 lockdowns have negatively impacted mental health in general population (10, 11) and quarantines have caused neuropsychiatric disorders in several parts of the world (12), which have worsened quality of life (QoL) (13, 14). QoL refers to an individual’s overall well-being and satisfaction with various aspects of their existence, including physical health, psychological state, social relationships, and living conditions (15). QoL encompasses subjective perceptions and objective measures that contribute to one’s overall life experience and sense of fulfillment. This QoL change can vary according to the characteristics of the population, which include family composition, as well as the quarantine period.

This study sought to assess the QoL of individuals, and families during the mandatory home quarantine related to the COVID-19 pandemic. A primary objective was to compare the results of QoL among countries during the 2020 COVID-19 outbreak. The research also aimed to understand the QoL between members of the family and inter-family’s QoL that were forced to live together under quarantine during the ongoing pandemic.

## 2. Materials and methods

### 2.1. Study design and settings

This study was cross-sectional based on online anonymous surveys administered in seven countries (Brazil, Colombia, Spain, Japan, Peru, Russia, and Venezuela). Between February 15 and May 30, 2020, countries worldwide enacted strategies to combat the COVID-19 pandemic. These efforts encompassed diverse containment and control measures, with the timing and effectiveness of responses varying across nations. As a result, disparities emerged in the societal reception and compliance with these interventions, driven by variations in the months when infection and mortality mitigation strategies were implemented (16, 17).

### 2.2. Sample and inclusion criteria

Employing a systematic approach, we utilized a straightforward random probabilistic sampling technique to ensure the diversity of our participant pool across various countries. The recruitment was conducted via prominent social networking platforms (i.e., Instagram, Facebook, and WhatsApp). To determine the appropriate sample size, we estimated a minimum of 278 participants, with a particular focus on achieving a minimum of 30 participants from each country. An open call was extended through these platforms to invite potential participants to take part in our study. Our inclusion criteria encompassed individuals aged >18 years, of both genders, who did not exhibit symptoms or a prior COVID-19 diagnosis. Participation was entirely voluntary, and we welcomed complete family units, including partners, mothers, and grandparents. However, families with at least one member diagnosed with COVID-19 and those with members under the age of 18 were excluded to maintain the clarity and homogeneity of the study population. The survey was administered through Google Forms (Google, CA, United States), distributed through two platforms of social networks: WhatsApp and Facebook. It is worth noting that no tangible incentives or rewards were provided to participants in exchange for their involvement in the study. Instead, participants were duly informed of the eventual outcome of the study, specifically regarding their QoL assessment. This ensured that their participation was driven by a genuine willingness to contribute to the research.

### 2.3. Procedure

Prior to their participation, all individuals involved in the study received comprehensive information regarding the study’s purpose and methodology. This communication was established through either a phone call or a video call, ensuring that participants were well-informed before proceeding. To further enhance transparency, participants were directed to an “Information Sheet for Participants and Families” through a provided link within the survey. This document elaborated on the study’s objectives and the benefits of their involvement. To accommodate a diverse group of participants, the surveys were made available in three languages: Spanish, Portuguese, and English. In alignment with legal standards, an informed consent process was meticulously adhered to, incorporating the provisions outlined in the Peruvian data protection law (No. 29,733) to safeguard participant privacy and rights (18). The study was conducted during the initial wave of the COVID-19 pandemic, spanning from February 15 to May 30, 2020. This period was carefully chosen to capture insights during a crucial phase of the pandemic, allowing us to gather data that would contribute to a comprehensive understanding of the impact on families’ QoL.

### 2.4. Measurement tool

We used the 26-item World Health Organization Quality of Life Brief (WHOQOL-BREF) questionnaire, which analyzes four components of QoL (physical, psychological, social relations, and environment) with five-item Likert-type scale (19). According to this survey, the highest scores indicate a better QoL in the two previous weeks. In addition, there were sociodemographic questions such as age, gender, and country of residence of the person who responded, family role, and employment (Table 1). These questions were defined by the opinions of research partners of the study of different countries, who reviewed and defined the final demographic questions. The Spanish version of the WHOQOL-BREF has been validated (Cronbach’s alpha = 0.88) (20, 21), as well as the Portuguese version (Cronbach’s alpha = 0.87 to 0.91) (22, 23), demonstrating robust reliability and internal consistency.

**Table 1:** Baseline characteristics of study participants.

Variables	Categories	*N* (%) or *N* (SD)
Gender	Male	168 (36.1)
	Female	298 (63.9)
Age (year)	Mean (SD)	38.59 (13.75)
	Median	33.89
	Range	18–83
	Range (IQR)	18–83 (19)
Work^b^	Yes	354 (76)
	No	112 (24)
Occupation/profession	Health professional	70 (15)
	Self-employed	70 (15)
	Management staff	38 (8.2)
	Education	36 (7.7)
	Others^a^	34 (7.3)
	Employee	34 (7.3)
	Housewife	30 (6.4)
	Laborer	30 (6.4)
	Engineer	14 (3)
	Retired	10 (2.1)
	Unemployed	70 (15)
Countries	Peru	162 (34.8)
	Colombia	68 (14.6)
	Russia	58 (12.4)
	Japan	58 (12.4)
	Venezuela	54 (11.6)
	Brazil	38 (8.2)
	Spain	28 (6)

a Includes police, architect, farmer, graphic designer, salesman, painter and merchant.

b Work in the last three months.

### 2.5. Outcomes, exposure and covariates

The impact of COVID-19 on quality of life in individuals and families from seven countries. Exposure to initial quarantine during the first wave of COVID-19. The covariates included the residence country, age, and gender of individuals, family relationship (role) between each participant and their relatives, type of employment/profession, and if he/she is currently working. All the covariates were based on self-report.

### 2.6. Missing data

There were no missing data, but three answers were eliminated for having been filled in incorrectly in regard to the covariates, and they were excluded of the analysis.

### 2.7. Statistical analysis

Initially, we performed descriptive statistics; this is, mean, standard deviation (SD), interquartile range (IQR) for all the variables. We used the Shapiro–Wilk and Kolmogorov–Smirnov tests to examine the normal distribution of the continuous variables. The results indicated that the required assumptions were not met for normal distribution and, because of that, data analysis used a non-parametric statistical method. *X*^2^ tests were used (when corresponded, Fisher’s exact tests) and Mann–Whitney *U* test to demonstrate differences among defined groups per each result. These comparisons included demographic characteristics (age, gender, occupation, family group, and country of residence) and QoL dimensions (physical, psychological, social relations, and environment). Finally, for the comparative analysis based on income categories, we referenced the latest World Bank report of 2022 (24). According to this report, Russia, Colombia, Peru, and Brazil fall under the category of upper middle-income countries [with gross national income (GNI) *per capita* ranging from $4,256 to $13,205], while Spain and Japan are classified as High-income countries (with GNI *per capita* of $13,206 or more). Venezuela is not included in this analysis as it does not have an income classification.

In addition, we used the Spearman’s correlation coefficient and we analyzed family group (defined by the roles of each individual in the family) in the QoL results. Family group was defined according to the family members (i.e., mother and son, grandmother and grandson). We performed the analyses among family groups and according to the six family types (i.e., nuclear, single-parent or blended) previously defined (25). For all tests, we considered a *p*-value of 0.05 and a 95% confidence interval statistically significant. The statistical analysis was performed in IBM SPSS V24.0 (Armonk, United States) and BoxPlotR (Tyers and Rappsilber Lab, Berlin, Germany) (26).

### 2.8. Ethical aspects

Ethics approval was granted by the Universidad Norbert Wiener Research Ethics Committee (Registry No. 2020-146-121-RRR-UNW).

## 3. Results

### 3.1. Demographic characteristics

There were 466 participants who belonged to 198 families in the seven countries. The average age was 38.59 ± 13.75 years old (ranging from 18 to 83), and 298 (63.9%) were female. In addition, 76% of the participants worked mostly as health professionals and self-employed (each 15%). Of the total, 168 were Peruvian, and 68 (14.6%) were Colombian. Demographic characteristics were shown in Table 1.

### 3.2. Quality of life during the confinement

**Figure 1:**
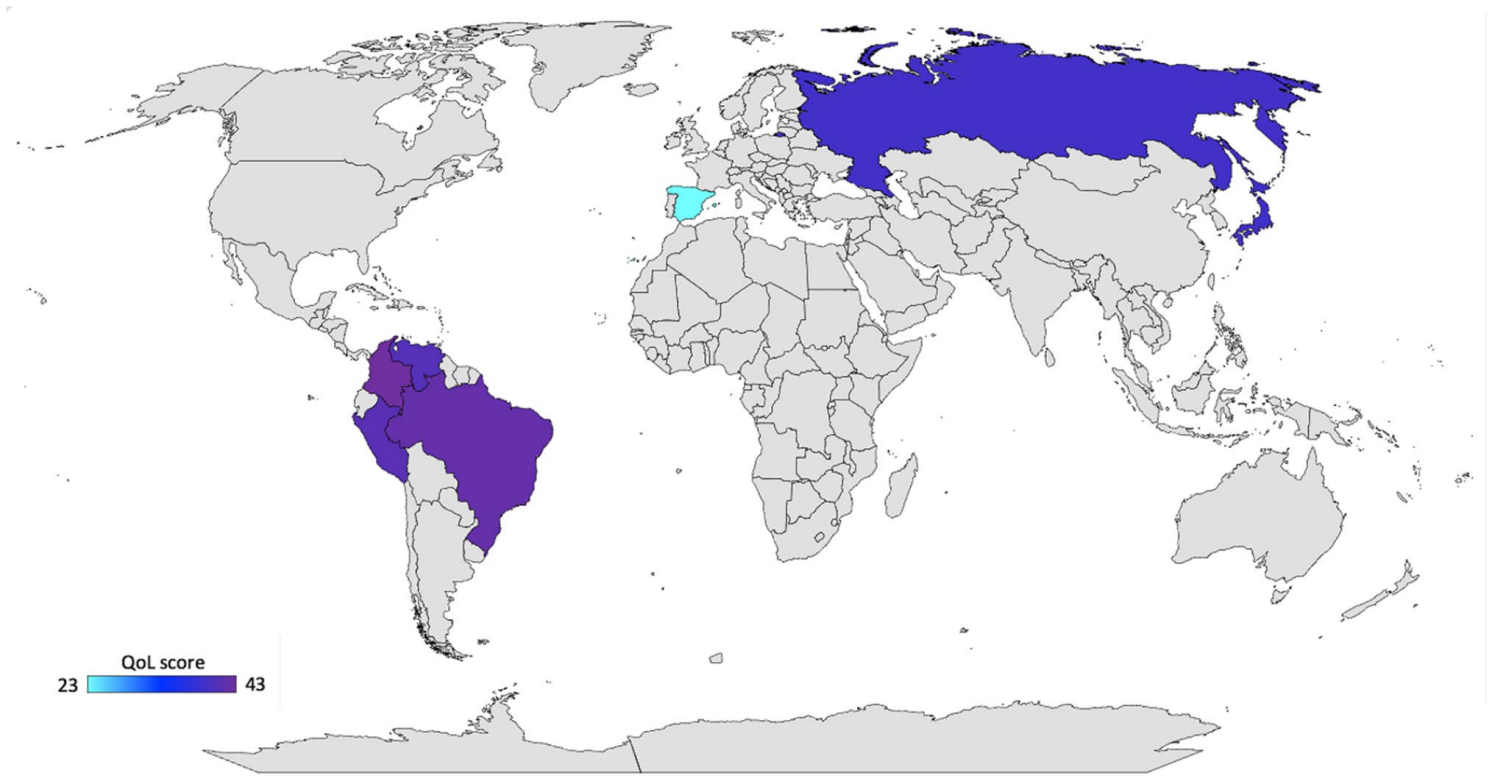
Global distribution of quality of life during the COVID-19 quarantine in 2020. Created by ^©^Jeel Moya-Salazar, Bing for Microsoft.

The average of QoL among the countries included in the study are shown in Figure 1. Overall average of QoL was 43 ± 7.72 (95% IC: 41.12 to 45.73) in Colombia; 42 ± 10.36 (95%IC: 39.08 to 45.66) in Brazil; 41 ± 7.63 (95% IC: 40.13 to 42.48) in Peru; 41 ± 9.20 (95% IC: 38.03 to 42.48) in Venezuela; 39 ± 8 (95% IC: 36.54 to 43.66) in Russia; 39 ± 6.88 (95% IC: 36.68 to 41.55) in Japan; and 23 ± 13.63 (95% IC: 17.23 to 28.37) in Spain (Table 2). We found significant differences between Peru and Brazil (*p* = 0.026).

**Table 2:** Multidimensional results of quality of life in the countries included in the study.

Variables	Categories	Quality of life domains				
		Physical	Mental	Social	Environmental	Global
Countries	Colombia	24 (6.12)	38 (7.26)	85 (20.79)	27 (6.13)	43 (7.72)
	Brazil	24 (8.57)	36 (8.65)	84 (24.65)	25 (8.72)	42 (10.36)
	Peru	21 (7.91)	37 (8.20)	86 (19.14)	22 (6.65)	41 (7.62)
	Venezuela	22 (9.37)	40 (8.39)	80 (24.98)	21 (6.1)	41 (9.20)
	Russia	18 (8.64)	34 (8)	84 (17.1)	20 (7.7)	39 (8)
	Japan	21 (5.53)	33 (7.82)	81 (18.31)	21 (5.67)	39 (6.88)
	Spain	13 (9.1)	14 (15.28)	41 (33.12)	23 (5.48)	23 (13.63)
*p*-value		0.142	0.053	0.047	0.221	0.304

Data in mean (SD).

**Figure 2:**
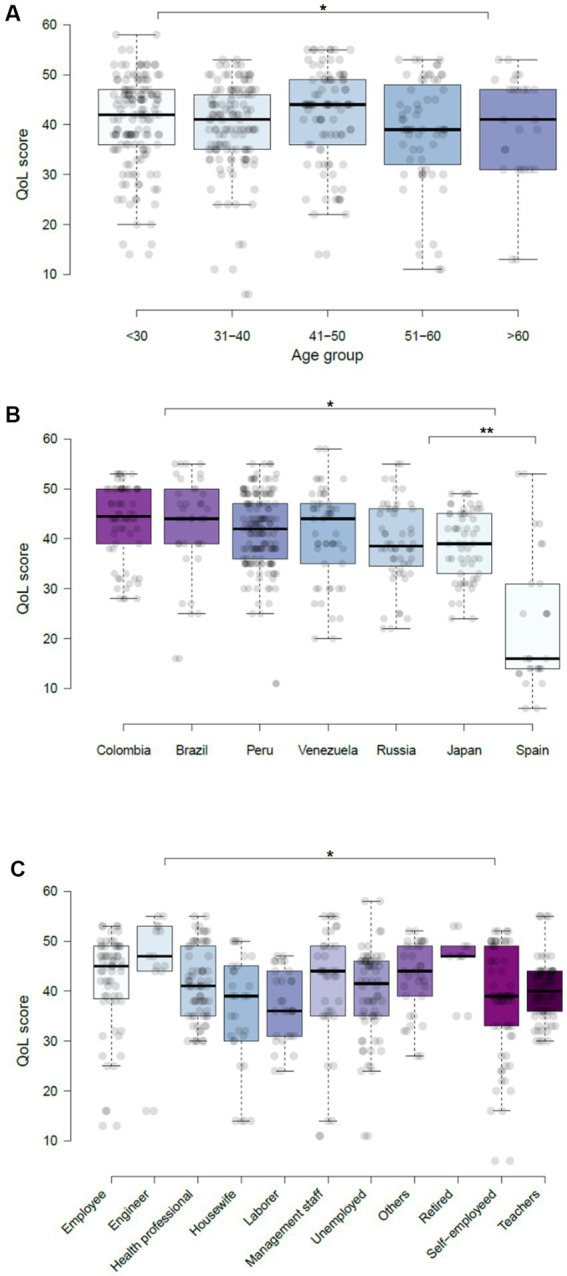
Global quality of life score according to demographic characteristics of the participants during COVID-19. **(A)** Age groups. **(B)** Country of origin. **(C)** Work. ^*^p < 0.05, ^**^p < 0.001.

When evaluating the QoL dimensions, we did not find differences in the scores by each dimension (*p* > 0.05) and determined that the social relations dimension was the only one with values close to 100 (average of 83.3) in 6/7 evaluated countries, where only Spain had a score of 41 ± 33.12. The physical, mental, and environmental dimensions were dramatically abolished with scores of 25, 41, and 28 QoL points, respectively. We found differences in the physical health dimension (*p* = 0.033) and mental health dimension of QoL between Spain and Japan, and, in addition, there were differences between Peru and Spain (*p* < 0.001) in the latter dimension. In the social relations dimension, we could evidence differences between Colombia and Brazil (*p* = 0.042), Spain and Venezuela (*p* = 0.009), and between Russia with Peru (*p* = 0.010) and Spain (*p* = 0.008). In regard to the environmental dimension, we only evidenced significant differences between Peru and Russia (*p* = 0.020) and Colombia (*p* = 0.010), (Figure 2B). Significant disparities in QoL were observed when comparing an Upper middle-income country with a High-income country [QoL: 41.2 (8.4) vs. 31 (10.25) points, *p* = 0.048].

### 3.3. Quality of life by gender and age

The dimensions of QoL according to the demographic characteristics are shown in Table 3. We did not find significant differences in the overall QoL (*p* = 0.354) of males (41.55 ± 9.98) and females (40.16 ± 8.78) included in the study (Supplementary Figure S1). The analysis by age has demonstrated the differences in the overall QoL between age group of 31–40 and >60 (*p* = 0.015) and <30 years *p* = 0.046. In regard to physical dimension, we only found differences among age groups of 31–40 and 51–60 years old (*p* = 0.045), while in the mental health dimension, we could find differences between age groups of <30 and 41–50 (*p* = 0.020), and also between 51–60 and >60 years old (*p* = 0.019). With respect to the social and environmental dimensions of QoL, we evidenced differences between age groups of 51–60 years old and >60 years old (*p* = 0.005), and between 41–50 years and >60 years (*p* = 0.02), respectively (Figure 2A).

**Table 3:** Demographic characteristics and multidimensional quality of life during the COVID-19 pandemic.

Variables	Categories	Quality of life domains				
		Physical	Mental	Social	Environmental	Global
Gender	Male	22.27 (7.74)	36.6 (9.92)	83.71 (23.23)	23.13 (6.82)	41.55 (9.98)
	Female	20.3 (7.83)	35.46 (7.64)	82.13 (20.72)	22.17 (7.39)	40.16 (8.78)
*p*-value		0.091	0.415	0.634	0.341	0.354
Age (year)	≤30	20.28 (8.57)	36.2 (9.33)	82.4 (22.88)	22.68 (6.66)	40.57 (9.1)
	31–40	19.6 (8)	33.95 (11.32)	81.1 (22.8)	22.21 (6.12)	39.33 (9.33)
	41–50	23 (7.94)	35.8 (9.99)	83.46 (23.13)	23.57 (8.41)	41.65 (9.74)
	51–60	21.4 (6.69)	34.25 (10.70)	77.37 (26.33)	20.62 (6.98)	38.5 (10.95)
	>60	25.1 (9.23)	33.15 (9.87)	75.46 (23.14)	24.15 (6.1)	39.5 (11.15)
*p*-value		0.096	0.213	0.155	0.401	0.239

Data in mean (SD).

### 3.4. Quality of life by type of work

The dimensions of QoL according to the characteristics of employment are shown in Table 4. QoL showed differences between engineers and health care workers (HCW) (*p* = 0.013), laborers (*p* = 0.002), employees (*p* = 0.011), and teachers (*p* = 0.037). We also found differences between HCW and unemployed (*p* = 0.037), housewives, and laborers (*p* = 0.001), and unemployed vs. self-employed (*p* = 0.007) (Figure 2C).

**Table 4:** Multidimensional results of quality of life according to working conditions.

Variables	Categories	Quality of life domains				
		Physical	Mental	Social	Environmental	Global
Work^b^	Yes	22.04 (6.9)	35.78 (7.98)	82.49 (19.18)	22.40 (7.73)	40.76 (8.41)
	No	19.38 (9.1)	31.35 (11.38)	75.11 (28.11)	22.02 (6.79)	37.1 (11.38)
*p*-value		0.092	0.009	0.163	0.797	0.073
Occupation/profession	Health professional	22.46 (7.8)	36.14 (9.29)	83.54 (17.49)	22.83 (7.53)	41.46 (7.22)
	Self-employed	20.1 (8.16)	34.26 (11.76)	74.29 (27.14)	22.23 (6.63)	37.83 (11.15)
	Management staff	20.89 (9.95)	35.37 (12.33)	80.63 (29.63)	24.84 (7.88)	40.63 (12.57)
	Education	19.72 (6.54)	35.16 (6.23)	83.61 (14.33)	21.1 (6.86)	40.1 (5.95)
	Others^a^	23.65 (6.9)	39.94 (7.18)	82.35 (22.52)	24.71 (5.84)	42.82 (7.31)
	Employee	22.28 (8.86)	24.69 (11.84)	86.91 (22.6)	22.31 (6.78)	41.72 (10)
	Housewife	19.27 (8.1)	29.67 (12.75)	71.27 (26.16)	22.6 (5.37)	35.73 (11.35)
	Laborer	17.8 (3.98)	37.7 (6.85)	80 (21.41)	18.6 (4.71)	37.1 (7.22)
	Engineer	28.86 (6.67)	38.43 (11.22)	85.71 (29.1)	26.71 (9.75)	44.57 (12.76)
	Retired	28.8 (8.8)	35 (5.53)	95 (10.54)	25.2 (10.54)	25.2 (7.31)
	Unemployed	18.94 (8.47)	35.56 (9.25)	80.68 (24.83)	21.59 (6.77)	39.35 (9.39)
*p*-value		0.102	0.370	0.490	0.553	0.277

Data in mean (SD).

a Includes police, architect, farmer, graphic designer, salesman, painter and merchant.

b Work in the last three months.

According to the analysis only in the mental health dimension (*p* = 0.009), we found differences among individuals who worked (Supplementary Figure S1). According to the type of work, we found differences in the physical health dimension between HCW compared to employees (*p* = 0.027), engineers (*p* = 0.006), and retired participants (*p* = 0.025). In addition, laborers showed significant differences compared to housewives (*p* = 0.001), and management staff (*p* = 0.038) (Figure 2C).

In regard to the mental health dimension, there were differences between employees when compared to unemployed (*p* = 0.012) and self-employed participants (*p* = 0.002), as well as HCW compared to management staff (*p* = 0.046) and self-employed (*p* = 0.048). Furthermore, the unemployed individuals had differences when compared to the self-employed ones (*p* = 0.004) and housewives (*p* = 0.048). On the other hand, we found differences between engineers compared to retired participants (*p* = 0.017), self-employed ones (*p* = 0.007), laborers (*p* = 0.048), and other professions (*p* < 0.001) in the social relations dimension. In this dimension, we also found differences between laborers compared to management staff (*p* = 0.043), teachers (*p* = 0.006), and housewives (*p* = 0.002), and self-employed compared to teachers (*p* = 0.007) and employees (*p* = 0.037). In the environmental dimension, we found differences between retired participants [compared to employees (*p* = 0.014) and other professions (*p* = 0.041)], self-employed compared to housewives (*p* = 0.010), and between unemployed and employees (*p* = 0.008).

### 3.5. Analysis of families under quarantine

**Figure 3:**
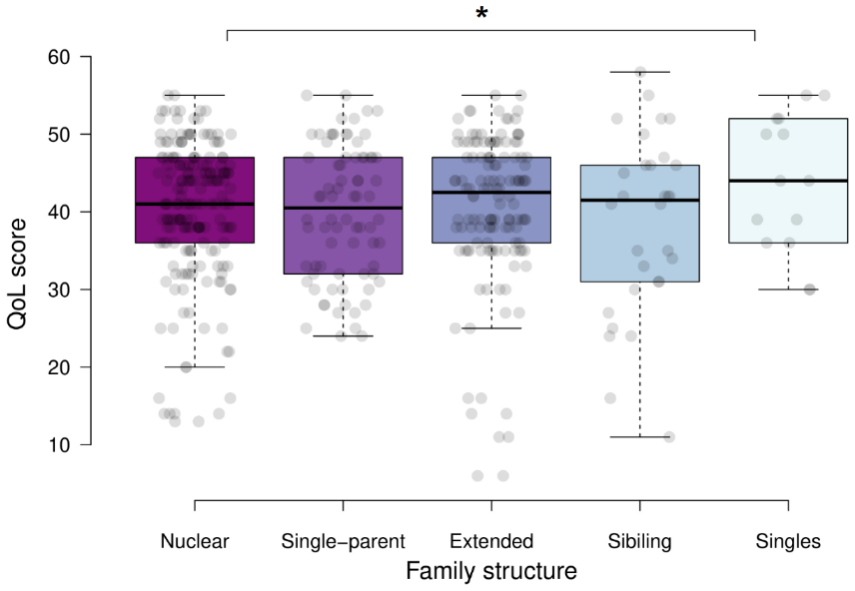
Global quality of life according to the structure of families under quarantine by COVID-19 in 2020. ^*^p < 0.05.

The results of the analysis by family structure are shown in Table 5. According to family structure, 72 (36.4%) were nuclear families, 50 (25.3%) were extended families, 26 (13.1%) were siblings, and 18 (9.1%) were single-parents. The overall QoL analysis only showed differences between nuclear families and siblings (*p* = 0.024). However, the analysis by dimension has demonstrated differences in the physical health dimension between nuclear families and siblings (*p* = 0.026), and extended families and single participants (*p* = 0.012). In the mental dimension, we found differences between siblings and singles (*p* = 0.045), and single-parent families and singles (*p* = 0.045). We did not find differences in the social and environmental dimensions (Figure 3).

**Table 5:** Multidimensional results of quality of life according to family structure.

Variables	Categories	Quality of life domains				
		Physical	Mental	Social	Environmental	Global
Family structure	Nuclear	20.81 (6.62)	34.79 (10.46)	82.48 (22.41)	22.08 (6.69)	40.04 (9.23)
	Single-parent	20.92 (7.31)	35.53 (7.45)	79.89 (20.89)	23.03 (7.83)	39.84 (8.68)
	Extended	20.97 (9.42)	33.52 (10.98)	84 (23.29)	22.48 (6.47)	40.24 (9.90)
	Sibling	20.77 (10.27)	36.77 (11.15)	73.50 (29.29)	22.37 (7.72)	38.35 (11.61)
Singles		27.43 (8.48)	43 (5.74)	76.86 (24.06)	26.86 (6.1)	43.54 (9.1)
*p*-value		0.815	0.163	0.506	0.134	0.550

*N* = 466.

## 4. Discussion

The international study, conducted during the first outbreak of COVID-19, showed a sharp drop in QoL in all seven countries. The countries most affected by COVID-19 are those with the lowest overall QoL (Japan in Asia, Russia and Spain in Europe), showing differences in maintaining high scores on the social dimension. In addition, subgroup analysis demonstrated that QoL was reduced regardless of family structure.

The main strength of the study is that, to the best of our knowledge, this is the first international study that has compared the QoL at the start of the pandemic in 2020 between countries in Europe and America. In addition, there are several reports (14, 27, 28) that have quantified the QoL of life during the pandemic but have not focused their analysis according to the type of family during the first outbreak of SARS-CoV-2, in this sense this study contributes to understanding the initial effects of COVID-19 on the quality of life of populations. Although mental health has been a recurring theme during the lockdown, some previous studies have not estimated the impact of the pandemic on QoL (20), while others have not delved into a multidimensional analysis of QoL (29–31). However, as in this study, the investigations that have used the WHOQOL-BREF have managed to delve into the components of the QoL and carry out a comprehensive assessment of its status (20–23, 32, 33).

Prolonged isolation, social distancing, and government policies can negatively impact QoL in populations (30, 34). Confinement can reduce the quality of sleep, daily physical activity, and social relationships, negatively impacting the well-being of the general population (35). Our results show that the impact of preventive measures, as well as the consequences of the pandemic in terms of deaths and daily infections, worsened the QoL following a geographic mark. In other words, where the peaks of infections occurred, the QoL was more lowered, as in Spain, while the countries with few cases or with the establishment of the lockdown due to COVID-19 had slightly higher levels. Although, all the countries assessed presented low levels of QoL (<50 points in WHOQOL-BREF), coinciding with studies in other countries (10, 11, 27, 29, 36).

Community indicators (infection rate) can affect and reduce QoL (33). In our study, we have seen that there is a marked reduction in countries with high rates of infections and deaths from COVID-19, although we did not show significant differences with countries with lower indicators of infection (South American countries). This may be explained at the multidimensional level of the QoL since interestingly the highest dimension was social relationships, even in Spain where the averages were low, and the pandemic had high peaks in infections and deaths, an average of 40 points in the QoL score. Social relationships have been seen to be maintained during confinement, and these relationships have been important vehicles for communication and emotional support worldwide, and as such, they have been preserved (32, 33, 37). During the period of restrictions established by the health emergency, populations must be protected and provided with truthful information and economic and social support (36). This issue is key to avoiding post-lockdown costs and problems that can cause a “wave” of damages after the aggressive event.

On the other hand, the imprint left by the pandemic in 2020 has also been affecting the well-being of its population, as the QoL remained low throughout the pandemic once it started (32, 38). As the COVID-19 outbreaks moved from Asia to Europe and the Americas, the QoL scores showed the effects of the pandemic on their low scores. The study was conducted at a time when European countries were being hit hard by COVID-19, which could explain the striking differences we found in mental, physical and environmental health. As the wake of the pandemic swept across Asia and Europe, a surge in mental health issues such as depression and anxiety ensued, precipitating a decline in the well-being of the affected population (39–41). In Latin America, the pandemic has triggered a substantial outbreak, prompting varied health responses with varying degrees of efficacy (17). These communities may face heightened mental health challenges due to a lack of culturally appropriate action policies (42, 43), which leads to significantly increased suicide risk has been reported (44). Indeed, the indigenous and rural Andean populations in Latin America have witnessed a reduction in their QOL, coupled with an upswing in mental health concerns such as anxiety, depression, and stress (38, 45). Further research should focus on comprehensively understanding the impact of COVID-19 on the well-being of populations worldwide.

Analysis of families revealed some differences in quality of life in terms of physical and mental health. Several studies have shown that QoL varies by family type (46), however, this study is the first of its kind to identify QoL levels in families in quarantine. The pandemic has affected families, mainly family leaders and women, by reducing their wellbeing (47). These results are consistent with our findings showing lower overall QoL among households. This family psychological distress may be exacerbated if your family members have chronic illnesses, as they experience greater psychiatric problems and lower quality of life during isolation (31). These effects have been seen in sibling family members (46) and will depend on family membership and pre-existing conditions. Consistent with our findings, several investigations have shown that women’s quality of life deteriorated during the pandemic (27, 29, 32, 36, 47). In addition, there are disparities between relatives in different age groups (8, 27, 30, 35), and we show differences in physical and mental health between young and old. These family characteristics are determinative of family well-being and may contribute to the persistence, reduction, or worsening of pre-existing conditions in certain sibling family members (31, 48–50).

This study had limitations. First, the generalizability of the findings was limited by participant selection bias, as only those with access to the virtual survey participated. While language was not an issue, as WHOQOL-BREF is available for research in several languages, samples were drawn voluntarily from each country and differences were found in the numbers of participants, which could have affected the conclusions of the study. Also, QoL is a concept that may be limited by survey understanding, but we were not able to assess it. Another limitation of this cross-sectional study is the inability to establish a causal relationship between the variables studied. This has prevented us from differentiating the effect of COVID-19 on pre-existing QoL, making it worse or better (38). Nonetheless, our inclusion of demographic and household data potentially facilitated participants’ accurate responses. Another aspect to consider is the temporal factor; the prevailing conditions related to COVID-19, as dictated by local and governmental measures (restrictions), might have influenced participants’ QoL responses. However, the effectiveness of the WHOQOL-BREF (19, 21–23) has managed to provide a quick overview of the QoL among families in the seven countries evaluated. Although the WHOQOL-BREF questionnaire is self-reported, this is one of the best instruments to assess QoL in the general population for clinical and research propose (51–53). Lastly, it is worth noting that a significant portion (around 76%) of participants were employed as health professionals, which could have conceivably impacted their questionnaire responses. The impact of the pandemic on healthcare workers has been profound, giving rise to a multitude of mental health challenges stemming from the overwhelming responsibilities, close interaction with patients, mobility constraints, and the persistent fear of contracting and transmitting the virus (2, 17, 54–56). While it is acknowledged that our findings may potentially bear the influence of these circumstances within the population, it’s crucial to underscore that our study also encompassed the assessment of QoL in family members. This broader scope allows us to contextualize well-being on a global over-individual scale. In the pursuit of a comprehensive understanding, it is imperative for future research to delve into a comparative analysis of our results with families not affiliated with healthcare professions. This comparative approach will provide valuable insights into the potential variations or parallels in QoL, thus contributing to a more nuanced comprehension of the factors at play.

## 5. Conclusion

Families from seven countries in Europe, Asia, and South America reported poor QoL during the first outbreak of COVID-19. This indicates that the pandemic prevention measures and disease burden impacted population well-being. However, the scores for social relations have been high, regardless of the social and epidemiological scenario of each country. In this sense, communication networks must also be key tools to face the debacle of the health of families in quarantine. This information can be used to inform international policymakers about how the deterioration of well-being during the lockdown has escalated to propose the development of personalized support services, adapted to each society and culture, and that cover the needs health of these families.

## Data availability statement

The raw data supporting the conclusions of this article will be made available by the authors, without undue reservation.

## Ethics statement

The studies involving humans were approved by Ethics approval was granted by the Universidad Norbert Wiener Research Ethics Committee (Registry No. 2020-146-121-RRR-UNW). The studies were conducted in accordance with the local legislation and institutional requirements. Written informed consent for participation in this study was provided by the participants’ legal guardians/next of kin.

## Author contributions

JM-S primarily carried out the study, wrote first drafts, and revised all documentation. JM-S, HC-P, and JM-E equally contributed to the design and supervision of the study and revised all study documentation, and the manuscript. EC-H, AJ-Q, and BC provided advice during the study and helped revise study documentation. EC-H, HC-P, and JM-S reviewed the manuscript. All authors contributed to the article and approved the submitted version.

## Conflict of interest

The authors declare that the research was conducted in the absence of any commercial or financial relationships that could be construed as a potential conflict of interest.

## Publisher’s note

All claims expressed in this article are solely those of the authors and do not necessarily represent those of their affiliated organizations, or those of the publisher, the editors and the reviewers. Any product that may be evaluated in this article, or claim that may be made by its manufacturer, is not guaranteed or endorsed by the publisher.
