# Supplementary figures and images for: Quality of life in families under quarantine: a cross-sectional study in seven countries during the first outbreak of COVID-19
Source: Front Psychiatry. 2023 Sep 5;14:1238569. doi: 10.3389/fpsyt.2023.1238569 (PMC10508986; doi:10.3389/fpsyt.2023.1238569)

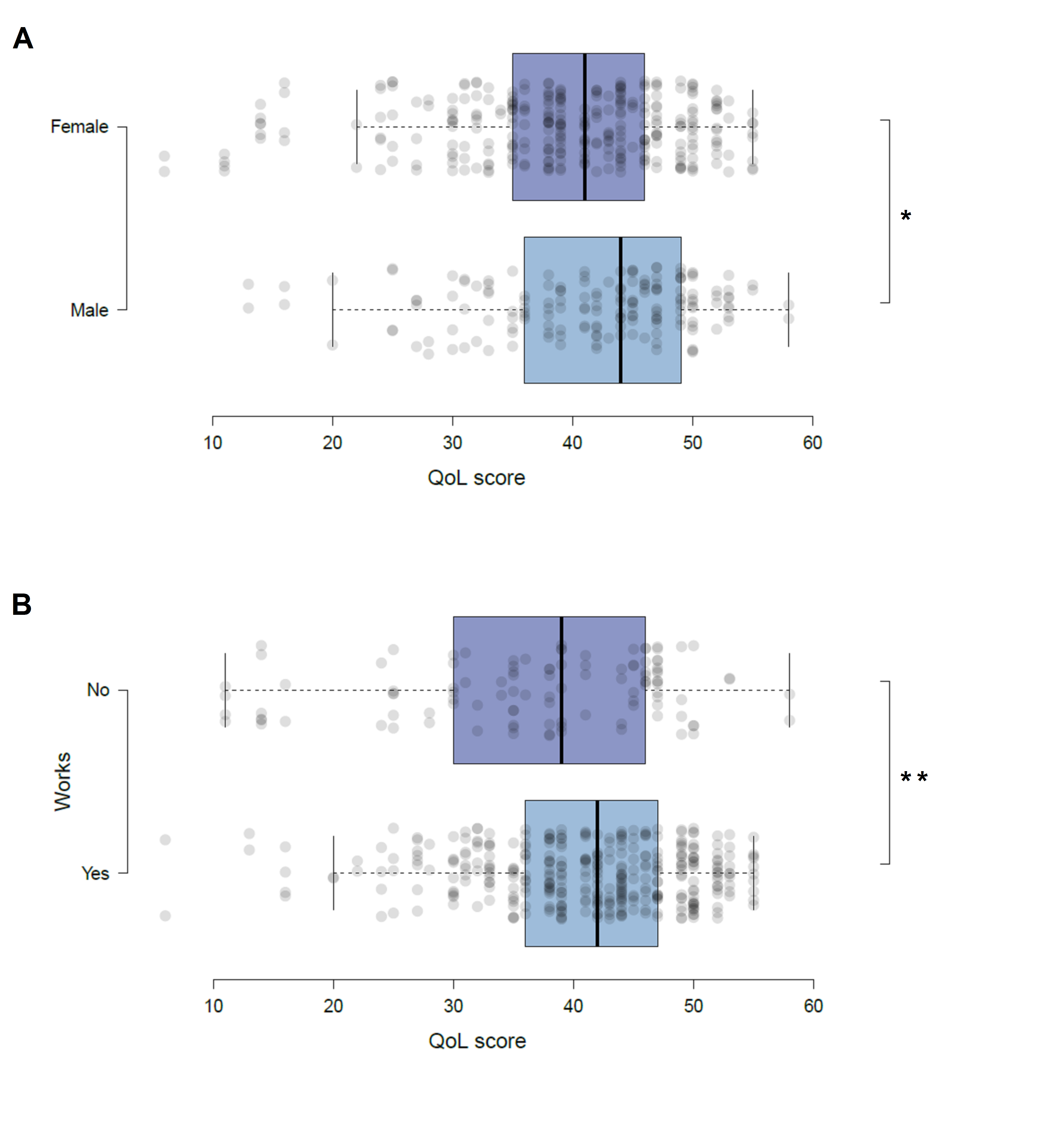

Supplement: Supplementary file 1 [file Image_1.TIF]
